# P-712. Effectiveness of Seasonal Influenza Vaccines against Medically Attended Influenza B, US Flu VE Network

**DOI:** 10.1093/ofid/ofae631.908

**Published:** 2025-01-29

**Authors:** Ashley Price, Jessie R Chung, Sascha Ellington, Brendan Flannery

**Affiliations:** Centers for Disease Control and Prevention, Mcdonough, Georgia; Centers for Disease Control and Prevention, Mcdonough, Georgia; CDC, Atlanta, Georgia; Centers for Disease Control and Prevention, Mcdonough, Georgia

## Abstract

**Background:**

Quadrivalent influenza vaccines including both B/Victoria and B/Yamagata components were introduced during the 2013-2014 season in the United States. With the worldwide interruption of influenza B/Yamagata virus circulation since March of 2020, trivalent vaccines including a B/Victoria component are expected to be recommended for the 2024-2025 season. We describe quadrivalent vaccine effectiveness (VE) against medically attended influenza B from 2016-2017 through 2019-2020 when the majority of the seasonal influenza vaccinations were quadrivalent.

**Methods:**

Study sites enrolled patients aged ≥6 months with acute respiratory illness and tested for influenza by RT-PCR. VE against influenza B was estimated using a test-negative design as 100% x (1 – adjusted odds ratio) comparing receipt of vaccination among B cases versus influenza-negative patients using logistic regression models adjusting for age, race/ethnicity, sex, calendar time and study site.

**Results:**

Over 4 seasons, 34,994 patients were enrolled, including 2,849 influenza B cases (1,507 [53%] type B/Yamagata and 1,342 [47%] B/Victoria), 7,539 influenza A cases (3,048 [40%] H1N1 and 4,491 [60%] H3N2) and 24,607 patients without influenza. Among influenza B cases, ratios of B/Yamagata to B/Victoria varied by season; no B/Yamagata cases were confirmed after 2020. By season, quadrivalent vaccine effectiveness among patients of all ages ranged from 48% to 55% against B/Yamagata and 56% to 85% against B/Victoria. VE tended to be highest among children aged 6 months – 8-years and against B/Victoria associated illness.

**Conclusion:**

Quadrivalent vaccines were effective against both lineages of influenza B viruses. Monitoring effectiveness of trivalent vaccines against B/Victoria and continued surveillance for emerging B lineages will be important.

**Disclosures:**

**All Authors**: No reported disclosures

